# The New Year

**Published:** 1917-01

**Authors:** 


### The New Year.

Unfortunately, a new year does not mean much to some people. The person who is trying to improve himself nearly always finds in it a time for reflection and resolutions; not resolutions to undertake the impossible, nor resolutions that are difficult to keep necessarily, but such resolutions as a person should keep in mind every day. The New Year merely furnishes a convenient starting place, the "tomorrow" on which so many of us are going to make a fresh start, but which never comes to many.

Every person must in this year 1917, as in the past, depend largely on himself for his successes and be responsible to himself for his failures. Others may assist to deter, but after all each of us is measured by what he does or himself. No person can rise above his ambitions and his efforts; no one can reckon himself a failure so long as he hopes and strives.

You have your problems and the TEXAS MEDICAL JOURNAL has its problems to meet and your success and ours are largely determined by the spirit with which we face the duties that will confront us. The MEDICAL JOURNAL is going to do its best for improvement, not for the mere sake of being better, but that it may render a better service, that it may do more for humanity through those who honor it by reading it. Doubtless you have something of the same thought in mind—you certainly have if you are worthy of the medical profession. That may appear to be an easy resolution to keep, but the only way it can be done is to strive to keep it not a year at a time, but a day at a time—to try to make each day more full than the day that has passed.

While we must each do for ourselves, we can help each other and are under the common obligation of humanity to do so. Co-operation is essential to progress in everything, and no one can live without it. You can't succeed in this day in cloistered seclusion, nor without helping others and in turn being helped.

Your success and ours depends in large measure upon how much of this spirit of helpfulness we show. The MEDICAL JOURNAL seeks it from every reader and in turn would manifest it to others. In other words, it prays your help in making a better JOURNAL, and wants to give back in good measure some help that you may need.

It is with something of these thoughts, however imperfectly they have been told, that the JOURNAL enters upon the New Year, and it is with the sincere wish that 1917 may be the best year that any of us have known, a wish that gives promise of rich fulfillment if only we will all strive earnestly enough to deserve it.

